# Synergistic Effects of Weighted Genetic Risk Scores and Resistin and sST2 Levels on the Prognostication of Long-Term Outcomes in Patients with Coronary Artery Disease

**DOI:** 10.3390/ijms23084292

**Published:** 2022-04-13

**Authors:** Hsin-Hua Chou, Lung-An Hsu, Jyh-Ming Jimmy Juang, Fu-Tien Chiang, Ming-Sheng Teng, Semon Wu, Yu-Lin Ko

**Affiliations:** 1 Division of Cardiology, Department of Internal Medicine, Taipei Tzu Chi Hospital, Buddhist Tzu Chi Medical Foundation, New Taipei City 23142, Taiwan; chouhhtw@gmail.com; 2 School of Medicine, Tzu Chi University, Hualien 97004, Taiwan; 3 Cardiovascular Division, Department of Internal Medicine, Chang Gung Memorial Hospital, Chang Gung University College of Medicine, Taoyuan 33305, Taiwan; hsula@adm.cgmh.org.tw; 4 Cardiovascular Center and Division of Cardiology, Department of Internal Medicine, National Taiwan University Hospital, Taipei 10002, Taiwan; jjmjuang@ntu.edu.tw (J.-M.J.J.); futienc@ntuh.gov.tw (F.-T.C.); 5 College of Medicine, National Taiwan University, Taipei 10051, Taiwan; 6 Cardiovascular Center and Division of Cardiology, Fu-Jen Catholic University Hospital, New Taipei City 24352, Taiwan; 7 Department of Research, Taipei Tzu Chi Hospital, Buddhist Tzu Chi Medical Foundation, New Taipei City 23142, Taiwan; vincent@tzuchi.com.tw (M.-S.T.); semonwu@yahoo.com.tw (S.W.); 8 Department of Life Science, Chinese Culture University, Taipei 11114, Taiwan

**Keywords:** resistin, soluble suppression of tumorigenicity 2, weighted genetic risk score, Taiwan Biobank, coronary artery disease, all-cause mortality, major adverse cardiac events

## Abstract

Resistin and soluble suppression of tumorigenicity 2 (sST2) are useful predictors in patients with coronary artery disease (CAD). Their serum levels are significantly attributed to variations in *RETN* and *IL1RL1* loci. We investigated candidate variants in the *RETN locus* for resistin levels and those in the *IL1RL1* locus for sST2 levels and evaluated the prognostication of these two biomarkers and the corresponding variants for long-term outcomes in the patients with CAD. We included 4652, 557, and 512 Chinese participants from the Taiwan Biobank (TWB), cardiovascular health examination (CH), and CAD cohorts, respectively. Candidate variants in *RETN* and *IL1RL1* were investigated using whole-genome sequence (WGS) and genome-wide association study (GWAS) data in the TWB cohort. The weighted genetic risk scores (WGRS) of *RETN* and *IL1RL1* with resistin and sST2 levels were calculated. Kaplan–Meier curves were used to analyze the prognostication of resistin and sST2 levels, WGRS of *RETN* and *IL1RL1*, and their combinations. Three *RETN* variants (rs3219175, rs370006313, and rs3745368) and two *IL1RL1* variants (rs10183388 and rs4142132) were independently associated with resistin and sST2 levels as per the WGS and GWAS data in the TWB cohort and were further validated in the CH and CAD cohorts. In combination, these variants explained 53.7% and 28.0% of the variation in resistin and sST2 levels, respectively. In the CAD cohort, higher resistin and sST2 levels predicted higher rates of all-cause mortality and major adverse cardiac events (MACEs) during long-term follow-up, but WGRS of *RETN* and *IL1RL1* variants had no impact on these outcomes. A synergistic effect of certain combinations of biomarkers with *RETN* and *IL1RL1* variants was found on the prognostication of long-term outcomes: Patients with high resistin levels/low *RETN* WGRS and those with high sST2 levels/low *IL1RL1* WGRS had significantly higher all-cause mortality and MACEs rates, and those with both these combinations had the poorest outcomes. Both higher resistin and sST2 levels, but not *RETN* and *IL1RL1* variants, predict poor long-term outcomes in patients with CAD. Furthermore, combining resistin and sST2 levels with the WGRS of *RETN* and *IL1RL1* genotyping exerts a synergistic effect on the prognostication of CAD outcomes. Future studies including a large sample size of participants with different ethnic populations are needed to verify this finding.

## 1. Introduction

Despite advancements in guideline-directed medical therapy, cardiovascular diseases remain the leading cause of disease burden and mortality worldwide. The global prevalence of cardiovascular disease nearly doubled from 271 million in 1990 to 523 million in 2019, and the number of cardiovascular deaths steadily increased from 12.1 million in 1990 to 18.6 million in 2019, half of which were due to ischemic heart disease [1]. To provide new insights into cardiovascular disease pathophysiology and to improve patient prognosis, investigators in the past two decades have focused on the association of numerous adipokines/cytokines with cardiovascular disease. Among these, resistin and suppression of tumorigenicity 2 (ST2) have attracted increasing interest in the past 10 years.

Resistin, belonging to a family of cysteine-rich proteins known as resistin-like molecules, was originally shown to be synthesized only by adipocytes and was demonstrated to induce insulin resistance in mice [2]. In humans, however, this protein appears to be expressed mainly by monocyte-derived macrophages [3]. As a proinflammatory adipokine in humans, resistin markedly upregulates the expression of inflammatory cytokines and cellular adhesive molecules [4,5]. Resistin appears to mediate the pathogenesis of atherosclerosis by promoting endothelial dysfunction, vascular smooth muscle cell proliferation, arterial inflammation, and foam cell formation [6,7]. It is predictive of atherosclerosis and poor clinical outcomes in patients with coronary artery disease (CAD), ischemic stroke, and congestive heart failure [7,8].

Approximately 70% of the observed variation in circulating resistin levels may be attributable to genetic factors [9], and several candidate genetic loci for resistin levels have been identified in different ethnic populations [9,10,11,12]. Genetic polymorphisms around the *RETN* locus are also related to resistin levels and various metabolic phenotypes, with discrepant results [11,12,13,14,15,16].

The ST2 receptor, a member of the interleukin (IL)-1 receptor family encoded by the IL-1 receptor-like 1 (*IL1RL1*) gene, is expressed as a membrane-bound receptor variant form (ST2L) and a truncated soluble form (sST2) [17]. IL-33, a biomechanically induced protein predominantly synthesized by cardiac fibroblasts, is the functional ligand of ST2L [18,19]. The IL-33/ST2 signaling pathway is upregulated in cardiomyocytes and fibroblasts in response to mechanical stimulation or injury and is cardioprotective in terms of reducing myocardial fibrosis, preventing cardiomyocyte hypertrophy, reducing apoptosis, and improving myocardial function [19,20]. By contrast, sST2 binds to IL-33 and acts as a “decoy” receptor for IL-33 to inhibit IL-33/ST2L signaling [19]. An elevated sST2 level is an independent predictor of subsequent mortality in patients with heart failure, acute myocardial infarction, and stable CAD [21,22,23,24,25].

*IL1RL1* variations can affect sST2 levels. In the genome-wide association study (GWAS) of the Framingham offspring cohort, up to 45% of the variation in sST2 levels not explained by clinical variables was attributed to genetic factors [26]. The most significant single nucleotide polymorphism (SNP) is rs950880, accounting for 12% of the individual variability in circulating sST2 levels. SNPs in the *IL1RL1* gene have also been linked to the severity of several immune and inflammatory diseases [27]. However, the effects of *IL1RL1* on predicting the outcome of cardiovascular disease remain unclear.

Our recent study indicated that individuals with the rs950880 AA genotype tended to have lower sST2 levels [28]; this genotype was an independent predictor of all-cause mortality in patients with CAD and lower-extremity arterial disease, and patients with high sST2 levels and the rs950880 AA genotype had the lowest survival rate. However, whether other biomarker levels can predict long-term outcomes in patients with CAD when combined with level-determining genotypes remains unknown. The Taiwan Biobank (TWB) conducted a large-scale population-based cohort study on 30–70-year-old volunteers with no history of cancer [29]. The genetic determinants of resistin and sST2 levels were derived from a regional association plot analysis using whole-genome sequence (WGS) data in a subgroup of 859 participants from the TWB cohort and from GWAS in 5000 participants from the TWB cohort. The weighted genetic risk scores (WGRS) of *RETN* and *IL1RL1* with resistin and sST2 levels were calculated using the data of independent level-determining genotypes. We hypothesized that WGRS of *RETN* and *IL1RL1* combined with both biomarker levels may better predict the long-term outcomes of patients with CAD.

## 2. Results

### 2.1. WGS Revealed Candidate SNPs in RETN and IL1RL1 Gene Loci

Given the previously reported *RETN* gene as the candidate locus for resistin levels, we first performed a regional association plot study with conditional analysis using data from 859 participants from the TWB cohort and the significance of resistin levels with 509 SNPs at positions between 7.715 and 7.755 Mb on chromosome 19p13.2 around the *RETN* gene region was assessed. Our data revealed that 17 SNPs exceeded the genome-wide significance threshold (*p* < 5 × 10^−8^), with rs3219175 being the lead SNP (*p* = 4.24 × 10^−72^) (Supplementary Figure S1A). To clarify whether the association of other *RETN* SNPs was independent of the lead SNP, we performed a stepwise conditional analysis. With adjustment for rs3219175, rs370006313 in the regional plot at the *RETN* locus became more significant with resistin levels (*p* = 6.56 × 10^−68^, Supplementary Figure S1B). Furthermore, with adjustment for both rs3219175 and rs370006313, rs3745368 remained significant (*p* = 1.24 × 10^−27^, Supplementary Figure S1C). With adjustment for all the three SNPs, none of the SNPs in the regional plot near the *RETN* locus exhibited significance at *p* < 0.01 (Supplementary Figure S1D), indicating that in this chromosomal region, variation in resistin concentrations was mainly explained by at least three signals.

We further evaluated the candidate gene variants for sST2 levels in the *IL1RL1* gene region using a regional association plot study with conditional analysis. In brief, 307 SNPs at positions between 102.915 and 102.985 Mb on chromosome 2q12.1 around the *IL1RL1* gene region were analyzed, and 242 SNPs exceeded the genome-wide significance threshold (*p* < 5 × 10^−8^), with rs6543115 being the lead SNP (*p* = 2.35 × 10^−85^) (Supplementary Figure S2A). With adjustment for rs6543115, rs1420091 in the regional plot at the *IL1RL1* locus became more significant with sST2 levels (*p* = 3.57 × 10^−15^, Supplementary Figure S2B). With adjustment for the two aforementioned SNPs, none of the SNPs initially showed genome-wide significance in the regional plot near the *IL1RL1* locus exhibited significance at *p* < 0.01 (Supplementary Figure S2C).

### 2.2. GWAS and Replication Genotyping Results for Resistin and sST2 Levels

**Figure 1:**
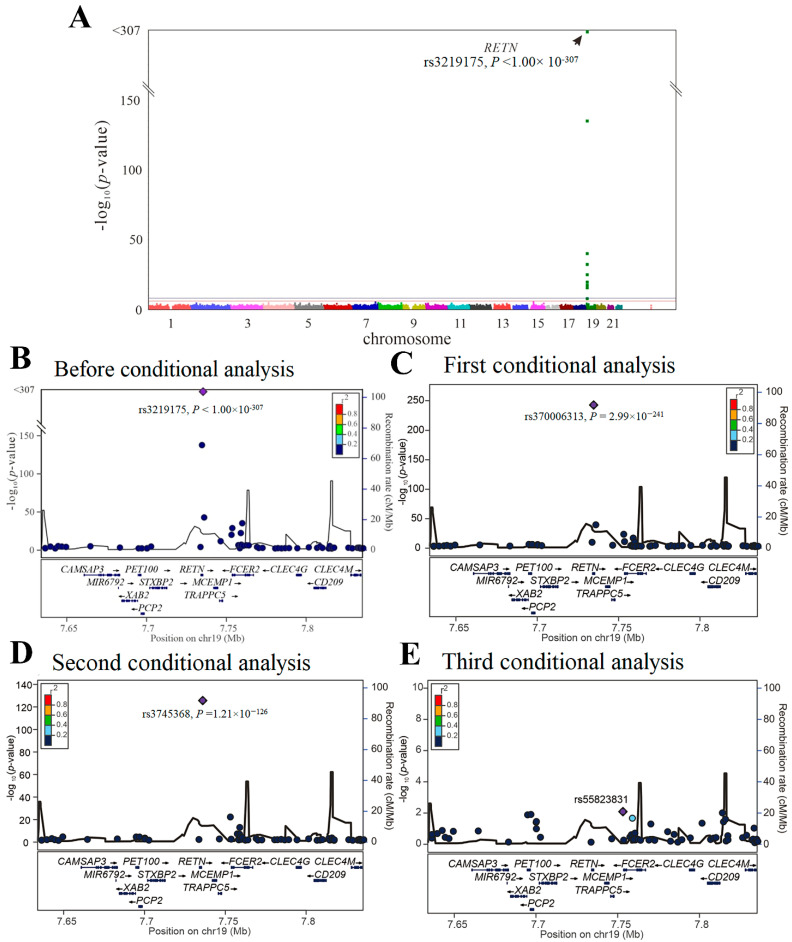
Conditional analysis of *RETN* candidate SNPs using GWAS data in the TWB cohort. (**A**) Manhattan plots for resistin levels from the genome-wide association study of 4652 Taiwan Biobank participants depict the only one peak above genome-wide significance on chromosome 19p13.2, where the *RETN* gene is located (arrow). (**B**) Before conditional analysis, regional association plots for resistin level surrounding the *RETN* locus show rs3219175 as the lead SNP. (**C**) After the first conditional analysis adjusting the rs3219175 genotypes, rs370006313 in the regional plot at the *RETN* locus becomes a significant association with resistin levels. (**D**) After the second conditional analysis adjusting for both rs3219175 and rs370006313 genotypes, rs3745368 is significantly associated with resistin levels. (**E**) After the third conditional analysis adjusting for the aforementioned SNPs, no more single SNP is found to be genome-wide significantly associated with resistin levels.

We then performed GWAS using the data of 4652 participants from the TWB cohort on a TWB genotype array for resistin levels. Because of the absence of the three lead aforementioned *RETN* SNPs (i.e., rs3219175, rs370006313, and rs3745368) in the TWB genotype array, we performed genotyping of the three *RETN* SNPs using TaqMan SNP Genotyping Assays. GWAS with conditional analysis indicated that the genome-wide significance threshold was exceeded only on chromosome 19p13.2, where *RETN* is located, and serial conditional analysis revealed the lead SNPs to be rs3219175, rs370006313, and rs3745368 (*p* < 1.00 × 10^−^^307^, *p* = 2.99 × 10^−241^, and *p* = 1.21 × 10^−126^, respectively) (Figure 1).

**Figure 2:**
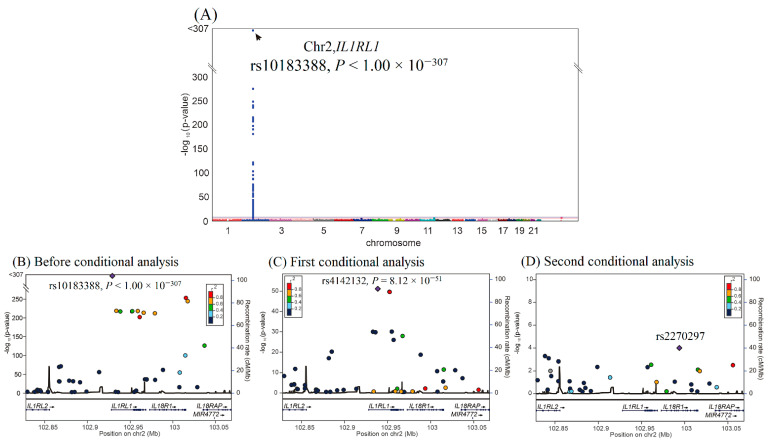
Conditional analysis of *IL1RL1* candidate SNPs using GWAS data in the TWB cohort. (**A**) Manhattan plots for sST2 levels from the genome-wide association study of 4652 Taiwan Biobank participants depict the only one peak above genome-wide significance on chromosome 2q12.1, where the *IL1RL1* gene is located (arrow). (**B**) Before conditional analysis, regional association plots for sST2 levels around the *IL1RL1* locus show rs10183388 as the lead SNP. (**C**) After the first conditional analysis adjusting for rs10183388 genotype, rs4142131 in the regional plot at *the IL1RL1* locus becomes a significant association with sST2 levels. (**D**) After the second conditional analysis adjusting for both rs10183388 and rs4142131, no more single SNP is found to be associated with sST2 levels significantly.

We then performed GWAS using the data of 4652 participants from the TWB cohort on a TWB genotype array for sST2 levels. GWAS with conditional analysis indicated that the genome-wide significance threshold was exceeded only on chromosome 2q12.1, where *IL1RL1* is located, and serial conditional analysis revealed that the lead SNPs were rs10183388 and rs4142132 (*p* < 1.00 × 10^−307^ and *p* = 8.12 × 10^−51^, respectively) (Figure 2). Further analysis revealed that rs10183388 and rs4142132 were in nearly complete linkage disequilibrium with the *IL1RL1* lead SNPs rs6543115 and rs1420091, respectively, in the WGS study (r^2^ = 0.98 and 0.96, respectively) (Supplementary Figure S3). The three lead *RETN* SNPs explained 53.7% of the variation in resistin levels, and the two lead *IL1RL1* SNPs explained 28.0% of the variation in sST2 levels (Supplementary Table S1)**.** The SNP table of *RETN* and *IL1RL1* is provided in Supplementary Table S2.

For the validation study, we analyzed the three lead *RETN* SNPs for resistin levels and two lead *IL1RL1* SNPs for sST2 levels in the cardiovascular health examination cohort (CH cohort). As shown in Table 1**,** the three *RETN* SNPs were significantly associated with resistin levels (rs3219175, rs370006313, and rs3745368 genotypes, *p* = 6.77 × 10^−41^, *p* = 1.36 × 10^−12^, and *p* = 2.70 × 10^−5^, respectively), and the two *IL1RL1* SNPs were significantly associated with sST2 levels (rs10183388 and rs4142132 genotypes, *p* = 9.11 × 10^−16^ and *p* = 2.73 × 10^−8^, respectively).

We evaluated the association of the *RETN* and *IL1RL1* SNPs with resistin and sST2 levels in the patients with CAD (Table 1), and the results revealed significant associations, except for rs370006313 genotypes for resistin levels (rs3219175, rs370006313, and rs3745368 genotypes for resistin levels, *p* = 4.89 × 10^−15^, *p* = 0.055, and *p* = 0.001, respectively, and rs10183388 and rs4142132 genotypes for sST2 levels, *p* = 4.49 × 10^−8^ and *p* = 8.00 × 10^−6^, respectively).

### 2.3. Baseline Characteristics of TWB, CH, and CAD Cohorts

The baseline characteristics of the participants in the TWB, CH, and CAD cohorts are provided in Table 2. The participants in the CAD cohort were older, with male predominance, and they tended to have a higher prevalence of smoking, obesity, hypertension, diabetes mellitus, and dyslipidemia. Compared with the TWB and CH cohorts, the CAD cohort had higher fasting glucose, triglyceride, aspartate aminotransferase (AST), and uric acid levels; higher leukocyte counts; lower total cholesterol, high-density lipoprotein (HDL)-cholesterol, and low-density lipoprotein (LDL)-cholesterol levels; and lower hematocrit, platelet counts, and estimated glomerular filtration rate (eGFR). The patients with CAD also had higher resistin levels but lower sST2 levels.

### 2.4. Correlations of Resistin and sST2 Levels with Clinical, Metabolic, Biochemical, Hematological Parameters, and WGRS of SNPs in All Cohorts

The correlations of resistin and sST2 levels with clinical, metabolic, biochemical, and hematological parameters and WGRS of SNPs are summarized in Supplementary Tables S3–S5. Resistin levels were strongly correlated with *RETN* WGRS in all three cohorts. Resistin levels were also significantly correlated with the lipid profile, eGFR, leukocyte count, and platelet count in the TWB cohort and with eGFR, leukocyte count, and hematocrit in the CAD cohort. Similarly, sST2 levels were strongly correlated with *IL1RL1* WGRS in all three cohorts. sST2 levels were significantly correlated with fasting glucose, lipid profile, AST, uric acid, eGFR, and leukocyte count in the TWB cohort and with eGFR and leukocyte count in the CAD cohort.

### 2.5. Association of Resistin Levels and RETN SNPs with Long-Term Outcomes for the Patients with CAD

**Figure 3:**
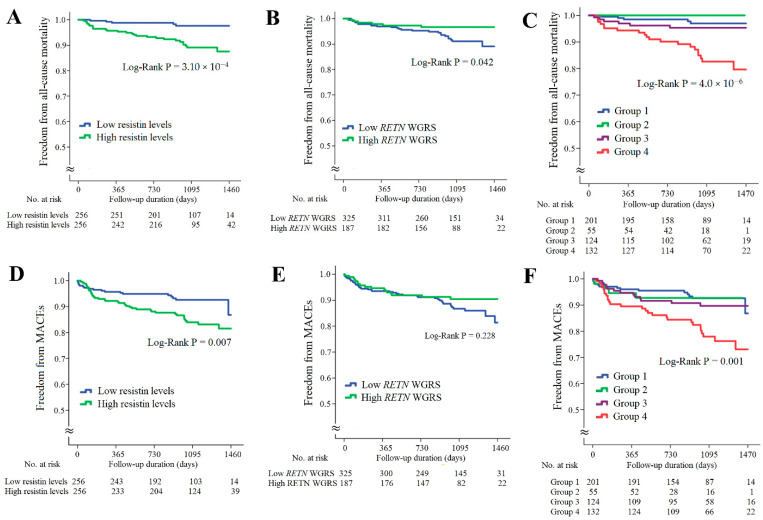
Kaplan–Meier curve analysis of resistin levels and *RETN* WGRS with long-term outcome in the patients with CAD. (**A**–**C**) Freedom from all-cause mortality in patients stratified by resistin levels, *RETN* WGRS, and combination of resistin levels and *RETN* WGRS. (**D**–**F**) Freedom from MACEs in patients stratified by resistin levels, *RETN* WGRS, and combination of resistin levels and *RETN* WGRS. WGRS, weighted genetic risk scores; MACEs, major adverse cardiac events. Group 1: low resistin levels/low *RETN* WGRS; Group 2: low resistin levels/high *RETN* WGRS; Group 3: high resistin levels/high *RETN* WGRS; Group 4: high resistin levels/low *RETN* WGRS.

In the CAD cohort, the follow-up duration was 1017 ± 324 days; 32 patients died and 58 patients developed major adverse cardiac events (MACEs). Kaplan–Meier survival analysis indicated that the patients with higher resistin levels had significantly higher rates of all-cause mortality and MACEs (Figure 3A,D, *p* = 3.10 × 10^−4^ and *p* = 0.007, respectively). The patients with lower *RETN* WGSR also had a higher rate of all-cause mortality, but *RETN* WGRS did not predict the MACEs rate (Figure 3B,E, *p* = 0.042 and *p* = 0.228, respectively). When the patients with CAD were further divided into four subgroups according to the resistin levels and *RETN* WGRS, the combination of high resistin levels and low *RETN* WGRS was a strong predictor of all-cause mortality and MACEs (Figure 3C,F, *p* = 4.0 × 10^−6^ and *p* = 0.001, respectively). The results of Cox regression analysis of all-cause mortality and MACEs between the groups stratified by the resistin levels and *RETN* WGRS are presented in Supplementary Table S6.

### 2.6. Associations of sST2 Levels and IL1RL1 SNPs with Long-Term Outcome for the Patients with CAD

**Figure 4:**
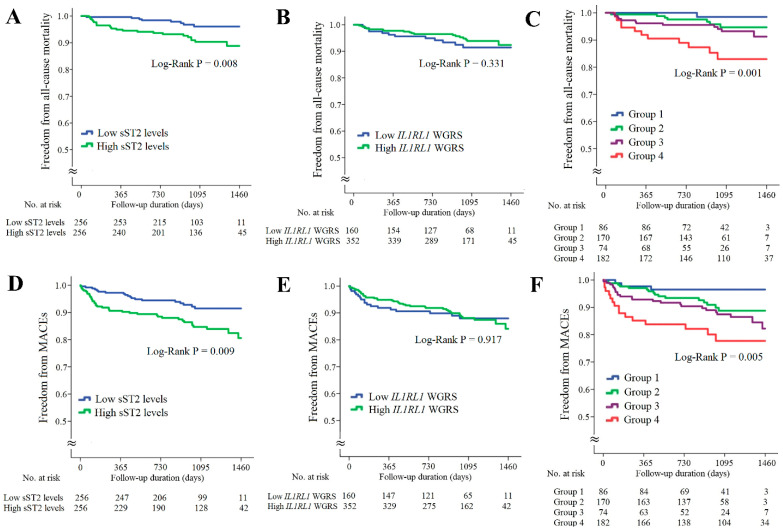
Kaplan–Meier curve analysis of sST2 levels and *IL1RL1* WGRS with long-term outcomes in the patients with CAD. (**A**–**C**) Freedom from all-cause mortality in patients stratified by sST2 levels, *IL1RL1* WGRS, and combination of sST2 levels and *IL1RL1* WGRS. (**D**–**F**) Freedom from MACEs in patients stratified by sST2 levels, *IL1RL1* WGRS, and combination of sST2 levels and *IL1RL1* WGRS. WGRS, weighted genetic risk scores; MACEs, major adverse cardiac events. Group 1: low sST2 levels/low *IL1RL1* WGRS; Group 2: low sST2 levels/high *IL1RL1* WGRS; Group 3: high sST2 levels/high *IL1RL1* WGRS; Group 4: high sST2 levels/low *IL1RL1* WGRS.

The associations of sST2 levels and *IL1RL1* SNPs with long-term outcomes for the patients with CAD are provided in Figure 4. The patients with higher sST2 levels had significantly higher rates of all-cause mortality and MACEs (Figure 4A,D, *p* = 0.008 and 0.009, respectively). However, the *IL1RL1* WGRS did not predict all-cause mortality and MACEs (Figure 4B,E, *p* = 0.331 and 0.971, respectively). When these patients were further divided into four subgroups according to the sST2 levels and *IL1RL1* WGRS, the combination of high sST2 levels and low *IL1RL1* WGRS was a strong predictor of all-cause mortality and MACEs (Figure 4C,F, *p* = 0.001 and 0.005, respectively). The results of Cox regression analysis of all-cause mortality and MACEs between the groups stratified by the sST2 levels and *IL1RL1* WGRS are provided in Supplementary Table S7.

### 2.7. Synergistic Effects of WGRS with Resistin and sST2 on Predicting Long-Term Outcomes of the Patients with CAD

**Figure 5:**
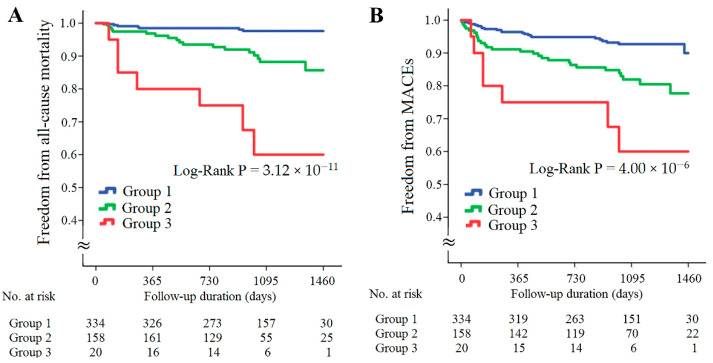
Kaplan–Meier curve analysis of combining resistin and sST2 levels with corresponding WGRS in predicting freedom from all-cause mortality (**A**) and freedom from MACEs (**B**) in the patients with CAD. WGRS, weighted genetic risk scores; MACEs, major adverse cardiac events. Group 1: patients without high resistin levels/low *RETN* WGRS and high sST2 levels/low *IL1RL1* WGRS; Group 2: patients with either high resistin levels/low *RETN* WGRS or high sST2 levels/low *IL1RL1* WGRS; Group 3, patients with both high resistin levels/low *RETN* WGRS and high sST2 levels/low *IL1RL1* WGRS.

The patients with CAD were further divided into three subgroups according to the presence of high resistin levels/low *RETN* WGRS or high sST2 levels/low *IL1RL1* WGRS (Figure 5). The patients with either high resistin levels/low *RETN* WGRS or high sST2 levels/low *IL1RL1* WGRS had significantly higher rates of all-cause mortality and MACEs, and the patients with both high resistin levels/low *RETN* WGRS and high sST2 levels/low *IL1RL1* WGRS had the worst outcomes (Figure 5A,B, *p* = 3.12 × 10^−11^ for all-cause mortality and *p* = 4.00 × 10^−6^ for MACEs). The results of Cox regression analysis of all-cause mortality and MACEs between the groups stratified by the presence of high resistin levels/low *RETN* WGRS and high sST2 levels/low *IL1RL1* WGRS are provided in Table 3. The patients with either high resistin levels/low *RETN* WGRS or high sST2 levels/low *IL1RL1* WGRS and the patients with both high resistin levels/low *RETN* WGRS and high sST2 levels/low *IL1RL1* WGRS had significantly higher rates of all-cause mortality and MACEs after adjustment for baseline characteristics and diseases such as hypertension, diabetes mellitus, or hyperlipidemia. However, predictive power was attenuated after further adjustment for uric acid level, eGFR, and inflammatory markers, including C-reactive protein (CRP), chemerin, and growth differentiation factor (GDF)-15 levels. Furthermore, significantly higher levels of inflammatory biomarkers and lower eGFR were observed in the patients with high resistin levels/low *RETN* WGRS or high sST2 levels/low *IL1RL1* WGRS (Supplementary Table S8).

## 3. Discussion

In this study, we performed two GWAS analyses for 4652 participants in the TWB cohort as well as regional association plot analyses for a subgroup of 859 participants with WGS data, aiming to elucidate the genetic basis of resistin and sST2 levels in the Chinese population. Three *RETN* variants (rs3219175, rs370006313, and rs3745368) and two *IL1RL1* variants (rs10183388 and rs4142132) were found to be independently associated with resistin and sST2 levels. In combination, these variants explained 53.7% and 28.0% of the variation in resistin and sST2 levels, respectively. These results were confirmed in validation studies in the CH and CAD cohorts. To the best of our knowledge, the three *RETN* variants were the strongest genetic determinants of resistin levels compared with the results of other GWAS analyses in ethnic population studies [13,14,15,30]. We further evaluated the effect of the combination of biomarker levels and genetic variants on predicting long-term outcomes in the patients with CAD. Both higher resistin and sST2 levels predicted higher rates of all-cause mortality and MACEs in the patients with CAD during long-term follow-up, but not WGRS of *RETN* and *IL1RL1* variants, except a borderline significance of low *RENT* WGRS on all-cause mortality. Notably, we observed a synergistic effect of the combination of biomarkers with WGRS of *RETN* and *IL1RL1* on the prediction of the long-term outcomes in the patients with CAD. The patients with high resistin levels/low *RETN* WGRS and the patients with high sST2 levels/low *IL1RL1* WGRS had significantly higher rates of all-cause mortality and MACEs. Furthermore, the patients with both high resistin levels/low *RETN* WGRS and high sST2 levels/low *IL1RL1* WGRS had the poorest outcomes over long-term follow-up.

### 3.1. Novel Variants Associated with Resistin Levels

GWAS of the data of 4652 participants in the TWB cohort revealed the *RETN* gene region as the only locus associated with resistin levels, with rs3219175 as the lead SNP (*p* < 1.00 × 10^−307^). This result confirmed the data from East Asian [11,12,16,30] and African populations [14] but was different from those reported in Caucasian populations, in which *TYW3/CRYZ* and *NDST4* loci, but not *RETN* variants, showed a genome-wide significant association with circulating resistin levels [10]. We used the GWAS data to analyze the *RETN* gene locus and found three *RETN* SNPs, namely rs3219175, rs370006313, and rs3745368, to be independently associated with resistin levels, which contributed to 53.7% of the variation in resistin levels in the TWB cohort. The data were replicated in the CH cohort (41.1% of variations). Notably, minor allele frequencies (MAFs) of two SNPs, rs3219175 and rs3745368, were significantly lower in the Caucasian population than that in East Asian and African populations [11,12,13,14,15]. By contrast, the MAF of the rs370006313 variant was 0.9% in our cohorts. According to the pubmed.gov website, the MAF of rs370006313 was 0% for 1006 European participants and 1322 African participants from the 1000-Genome project. Furthermore, the *RETN* –420C > G (rs1862513) genotypes, initially considered candidate SNPs for resistin levels [9,11,12,16], were not found to be independent determinants of resistin levels in our study population.

These results indicate ethnic heterogeneity not only at the level of gene loci but also at the level of each gene variant. rs370006313 is a novel SNP that is localized within the minimal promoter of human *RETN,* and it has not been previously reported to be associated with resistin levels. Although rs370006313 is a rare variant in the Taiwanese population, the resistin level increased by 4.7 times in the heterozygous state; thus, this SNP contributed to a 13.8% variation in resistin level in the relatively healthy population. Further research is warranted to elucidate the functional significance of this variant.

### 3.2. Genetic Determinants of sST2 Levels in Taiwan

In the GWAS analysis, our data revealed that chromosome 2q12.1, where the *IL1RL1* gene is located, was the only gene locus associated with sST2 levels. Further GWAS analysis revealed that the two lead *IL1RL1* SNPs, rs10183388 and rs4142131, were independently associated with sST2 levels and contributed to 28% of the variation; thus, these SNPs are strong genetic determinants of sST2 levels in the Chinese population. Consistently, the Framingham offspring cohort study reported that all candidate SNPs associated with the sST2 concentration were on chromosome 2q12.1 and that the 11 “independent” genome-wide significant SNPs across the *IL1RL1* locus accounted for 36% of the heritability of sST2 levels, with rs950880 as the lead SNP [26]. In the TWB cohort, GWAS analysis also revealed a significant association of rs950880 with sST2 levels (*p* = 1.73 × 10^−216^). Notably, this association disappeared after serial conditional analysis with rs10183388 and rs4142131, suggesting ethnic heterogeneity in *IL1RL1* variants.

### 3.3. Association of Resistin Levels with Cardiometabolic Phenotypes and Long-Term Outcomes in the Patients with CAD

As a proinflammatory adipokine, resistin is associated with several cardiometabolic phenotypes, including obesity, diabetes, and hyperlipidemia [31,32]. Elevated resistin levels are also associated with impaired renal function and several inflammatory biomarkers [33,34]. Resistin may also contribute to cholesterol and triglyceride accumulation in macrophages, arterial inflammation, endothelial dysfunction, and angiogenesis [31], leading to accelerated atherogenesis and CAD. In line with these findings, our study also found a strong association of resistin levels with BMI, diabetes mellitus, dyslipidemia, impaired renal function, and elevated leukocyte counts in the TWB cohort. Furthermore, elevated resistin levels were a strong predictor of poor long-term outcomes in the patients with CAD, and the participants with higher resistin levels had significantly higher rates of all-cause mortality and MACEs.

### 3.4. Association of sST2 Levels with Cardiometabolic Phenotypes and Long-Term Outcome in the Patients with CAD

sST2, a protein secreted by cultured myocytes subjected to mechanical strain, is a well-known biomarker of severe heart failure and a strong predictor of mortality [21,22]. IL-33, the ligand of sST2, is also induced and released by stretched myocytes. In patients presenting to the emergency department with myocardial infarction with ST elevation and dyspnea, sST2 levels are strongly predictive of heart failure and mortality [23]. In patients with stable CAD, increased sST2 levels also predict long-term MACEs and all-cause mortality [24,25]. Recently, sST2 levels have also been found to be a novel biomarker and clinical predictor of metabolic syndrome. In patients without CAD or heart failure, Zong et al. reported that increased sST2 levels were significantly associated with a higher prevalence of hypertension, diabetes mellitus, hypertriglyceridemia, and lower HDL cholesterol levels [35]. Consistently, in the present study, elevated sST2 levels were associated with a higher prevalence of diabetes mellitus, higher BMI, total cholesterol, and lower HDL cholesterol levels in the TWB cohort. Furthermore, elevated sST2 levels were significantly associated with higher AST, uric acid, leukocyte counts, and lower eGFR. For the patients with CAD, elevated sST2 levels predicted poor long-term clinical outcomes. The patients with higher sST2 levels had higher rates of all-cause mortality and MACEs during the follow-up period.

### 3.5. RENT and IL1RL1 Variants on Long-Term Outcomes in the Patients with CAD

The results of the association between *RENT* variants and CAD risk were inconsistent in previous studies. The association of *RETN* –420C > G with the risk of CAD was not significant in the Caucasian population [36,37]. By contrast, Tang et al. evaluated the association of *RETN* –420C > G with the presence of CAD and reported a 62% increased risk of CAD in participants with variant genotypes (CG and GG) [38]. Together, these findings suggest the ethnic heterogeneity of *RETN* variants with the risk of CAD. Importantly, none of these studies evaluated the prognostication ability of *RETN* variants for CAD outcomes. In the present study, the patients with CAD with low *RETN* WGRS had an increased all-cause mortality rate during the 4-year follow-up, but low *RETN* WGRS did not predict a high MACEs rate.

The evidence of the association between *IL1RL1* variants and the risk of CAD remains limited. In a case-control association analysis of 4521 individuals with CAD and 4809 controls in the Chinese Han population, Tu et al. reported a strong association of the *IL1RL1* SNP rs11685424 with the risk of CAD and disease severity [39]. Our previous study reported that among patients with CAD and lower-extremity arterial disease, those with the *IL1RL1* SNP rs950880 AA genotype tended to have lower sST2 levels and a lower survival rate [28]. The present study further evaluated the effect of *IL1RL1* variants on the prediction of long-term outcomes in the patients with CAD using the WGS and GWAS data from the TWB cohort. However, the WGRS of the two *IL1RL1* lead SNPs did not predict the outcomes of the patients with CAD, including all-cause mortality and MACEs.

### 3.6. Synergistic Effects of Genetic Variants and Resistin and sST2 Levels on Predicting Long-Term Outcomes in the Patients with CAD

When we combined the biomarkers with the WGRS of *RETN* and *IL1RL1*, we observed a synergistic effect on predicting long-term outcomes in the patients with CAD. The patients with high resistin levels/low *RETN* WGRS had the highest all-cause mortality and MACEs rates during the follow-up period (6.2 times for all-cause mortality and 3.1 times for MACEs compared with patients with low resistin levels/low *RETN* WGRS, Figure 3 and Supplementary Table S6). In agreement with this finding, the patients with high sST2 levels/low *IL1RL1* WGRS also had the poorest prognosis during the follow-up period (14.1 times for all-cause mortality and 6.0 times for MACEs compared with the patients with low sST2 level/low *IL1RL1* WGRS, Figure 4 and Supplementary Table S7).

We further analyzed the synergistic effects of combining resistin levels with *RETN* WGRS and sST2 levels with *IL1RL1* WGRS in the prognostication of long-term outcomes in the patients with CAD. The patients with high resistin levels/low *RETN* WGRS or high sST2 levels/low *IL1RL1* WGRS had 5.0 times higher all-cause mortality and 2.6 times higher MACEs rates than those without these presentations (Table 3). Furthermore, the patients with both high resistin levels/low *RETN* WGRS and high sST2 levels/low *IL1RL1* WGRS had the poorest outcomes (19.6 times for all-cause mortality and 5.9 times for the MACEs). These results were further adjusted for traditional cardiovascular risk factors, including age, sex, BMI, smoking status, diabetes mellitus, hypertension, and dyslipidemia, and adjusted for known predictors of poorer cardiovascular outcomes, such as uric acid levels [40], eGFR [41], and inflammatory biomarkers. These findings remained unchanged after adjustment for traditional cardiovascular risk factors but were attenuated after further adjustment of uric acid levels, eGFR, and inflammatory markers such as CRP, chemerin, and GDF-15 levels. Furthermore, significantly higher levels of CRP, chemerin, and GDF-15 and lower eGFR were observed in the patients with high resistin levels/low *RETN* WGRS and/or high sST2 levels/low *IL1RL1* WGRS (Supplementary Table S8). These findings indicate that inflammation and impaired renal function may explain the poorer outcomes in the patients with high biomarker levels and low WGRS for the corresponding genetic variants.

### 3.7. Study Limitations

Although we had enrolled a large sample size with available WGS and GWAS data in the TWB cohort, the sample sizes in the validation group (CH cohort) and study group (CAD cohort) were relatively small. However, the candidate SNPs in the *RETN* and *IL1RL1* genes were consistent in the three groups, providing strong evidence of the lead SNPs for *RETN* and *IL1RL1* in the Chinese population. Second, the event rates of all-cause mortality and MACEs were low in the CAD cohort. Nevertheless, we found a significant prediction power of resistin and sST2 levels for outcomes in patients with CAD, as well as a synergistic effect when the resistin and sST2 levels were combined with the WGRS of *RETN* and *IL1RL1*. Future studies should include a large sample size of patients with CAD to verify this finding. Finally, because we only included individuals of Han Chinese ethnicity, our results cannot be extended to other ethnic groups.

## 4. Materials and Methods

### 4.1. Study Populations

#### 4.1.1. TWB Cohort

The study cohort for the GWAS comprised 5000 participants from the TWB cohort, and WGS data were available for 859 of them. The data were collected from recruitment centers across Taiwan between 2008 and 2015. The inclusion criteria were participants without a history of cancer, stroke, CAD, or systemic disease. All participants self-reported having Han Chinese ethnicity. After participants were excluded based on the exclusion criteria, 4652 participants remained and were included in the GWAS (Supplementary Figure S4). Supplementary Table S9 shows the definitions of hypertension, diabetes mellitus, hyperlipidemia, and current smoking status. The Research Ethics Committee of Taipei Tzu Chi Hospital (approval number: 05-X04-007), Buddhist Tzu Chi Medical Foundation, and Ethics and Governance Council of the Taiwan Biobank (approval number: TWBR10507-02 and TWBR10611-03) approved our study. Written informed consent was obtained from all participants before participation.

#### 4.1.2. CH Cohort

The validation group was recruited during routine cardiovascular health examinations from October 2003 to September 2005 at Chang Gung Memorial Hospital and comprised 617 Han Chinese participants (327 men with a mean age of 45.2 ± 10.5 years and 290 women with a mean age of 46.8 ± 10.1 years), who responded to a questionnaire on their medical history and lifestyle characteristics. A total of 60 participants were excluded from the current study, and 557 participants were enrolled in the analysis (Supplementary Figure S4). All of the participants provided written informed consent, and the study was approved by the Ethics Committee of Chang Gung Memorial Hospital and the Ethics Committee of Taipei Tzu Chi Hospital, Buddhist Tzu Chi Medical Foundation.

#### 4.1.3. CAD Cohort

In the CAD cohort, 565 patients who received coronary angiography, had at least 50% stenosis of one major coronary artery, and had available blood samples for DNA and biomarker analyses were recruited between July 2010 and September 2013 from National Taiwan University Hospital. Among these, 53 patients were excluded from the current study, and 512 patients were included in the analysis (Supplementary Figure S4). All clinical data were obtained from the patients’ medical records. The primary enpoint was all-cause mortality. The secondary endpoint was MACEs, including the composite endpoints of all-cause mortality, hospitalization for heart failure, nonfatal myocardial infarction, or nonfatal stroke. Seven patients who were lost to follow-up after enrollment were contacted by telephone before the end of the study. Three of these patients had died, and the cause of death was provided by the relatives. All of the participants provided written informed consent, and the study was approved by the Research Ethics Committee of National Taiwan University Hospital.

### 4.2. Laboratory Examination

We examined the following clinical phenotypes: body height, body weight, body mass index (BMI), and systolic, mean, and diastolic blood pressure. In addition, we collected the following biochemical data: lipid profile, including total cholesterol, HDL cholesterol, LDL cholesterol, and triglyceride levels; fasting plasma glucose; and liver and renal functional test-related parameters such as serum creatinine, eGFR, uric acid, and AST. Hematological parameters included white and red blood cell counts, platelet counts, and hematocrit. Circulating levels of resistin, sST2, and inflammatory markers such as chemerin and GDF-15 were measured using commercially available enzyme-linked immunosorbent assay kits (R&D, Minneapolis, MN, USA). Circulating plasma levels of CRP were measured using the particle-enhanced turbidimetric immunoassay technique (Siemens Healthcare Diagnostics, Camberley, UK). The increase in turbidity that accompanies aggregation is proportional to the CRP concentration. Overall, the intra- and interassay coefficients of variation were 1.2–9.5% (Supplementary Table S10).

### 4.3. Genomic DNA Extraction and Genotyping

DNA of the participants was isolated from blood samples using a QIAamp DNA blood kit following the manufacturer’s instructions (Qiagen, Valencia, CA, USA). SNP genotyping was conducted using custom TWB chips and was run on the Axiom Genome-Wide Array Plate System (Affymetrix, Santa Clara, CA, USA). Genotyping for *RETN* rs3219175, rs370006313, and rs3745368 genotypes in the participants from the TWB cohort, the participants from the CH cohort, and the patients with CAD as well as for *IL1RL1* rs10183388 and rs4142132 genotypes were performed using TaqMan SNP Genotyping Assays (Applied Biosystems, Foster City, CA, USA). For quality control purposes, approximately 10% of the samples were re-genotyped blindly, and identical results were obtained.

### 4.4. TWB Whole-Genome Sequencing: RETN and IL1RL1 Gene Region

The WGS data of 859 participants from the TWB cohort were evaluated using an ultra-fast whole-genome secondary analysis on Illumina sequencing platforms [42] (Illumina HiSeq 2500/4000). The resulting reads were aligned to the hg19 reference genome with iSAAC 01.13.10.21. iSAAC Variant Caller 2.0.17 was used to perform SNP and insertion-deletion variant discovery and genotyping [42]. An in-house protocol written in shell script was performed to combine 880 vcf files. A union table of all detected variants in 880 vcf files was used for further analysis. Two gene loci linked to resistin and sST2 levels, according to the GWAS results, were included in the analysis, namely 20 Kb around the *RETN* and *IL1RL1* gene regions. The association between SNPs and resistin and sST2 levels was then analyzed using the GWAS method.

### 4.5. GWAS Analysis

The Axiom Genome-Wide CHB 1 Array Plate (Affymetrix) designed by the Taiwan Biomarker Study Group is a TWB genotype array used for GWAS analysis. In this genotyping platform, SNPs with minor allele frequencies of ≥5% in a set of 1950 samples were selected from Taiwan Han Chinese populations previously genotyped at the National Center of Genome Medicine of the Academia Sinica, Taipei, Taiwan [43]. Each genomic DNA was genotyped on the Axiom TWB genome-wide array comprising 642,832 SNPs with assistance from the National Center of Genome Medicine of Academia Sinica. All the samples in the analysis had a call rate of ≥97%. For SNP quality control, SNP call rate < 97%, minor allele frequency <0.01, and violation of Hardy–Weinberg equilibrium (*p* < 10^−^^6^) were criteria for exclusion from subsequent analyses. In total, 4652 participants and 614,821 SNPs were included in the GWAS analysis after quality control.

### 4.6. Statistical Analysis

Before analysis, resistin and sST2 levels were logarithmically transformed to adhere to a normality assumption. A generalized linear model was used to analyze resistin and sST2 levels in relation to the investigated genotypes and confounders. We assumed the genetic effect to be additive after adjustment for age, sex, BMI, and current smoking status. The software package PLINK was used to conduct genome-wide scans, and *p* < 5 × 10^−8^ was considered genome-wide significant. For GWAS, a conditional analysis was conducted to assess the residual association with all remaining SNPs after adjustment for the most strongly associated SNP at a locus by adding the SNP as a covariate into the regression model.

The baseline characteristics of all participants were evaluated using analysis of variance. Continuous variables are expressed as mean ± standard deviation or median and interquartile ranges when the distribution is strongly skewed. Differences in categorical data distribution were examined using a chi-square test or a chi-square test for trends. The Bonferroni method was used for post-hoc analysis after ANOVA. The genetic risk score was calculated using the weighted method, which assumed each SNP to be independently associated with resistin or sST2 levels (i.e., no interaction between each SNP) [44]. We assumed an additive effect of risk alleles for each SNP and applied linear weighting of 0, 1, or 2 to genotypes containing a corresponding number of risk alleles. WGRS were calculated by multiplying the estimated beta-coefficient of each SNP by the number of corresponding risk alleles (0, 1, or 2). Pearson’s correlation coefficients were used to examine the relationship of resistin and sST2 levels with clinical and biochemical factors in addition to WGRS. Each variable with a significant association with resistin and sST2 was entered into the multivariate linear regression model with the stepwise method to identify the independent correlates of resistin and sST2.

The rates of freedom from primary and secondary endpoints in the patients with CAD, stratified by the levels and resistin and sST2 and the WGRS of *RETN* and *IL1RL1* genotyping, were assessed using Kaplan–Meier curves and compared using the long-rank test. The outcomes were further analyzed by combining WGRS according to *RETN* and *IL1RL1* genotyping with both biomarker levels. Cox regression analysis was used to determine the hazard ratio of primary and secondary endpoints in each group. IBM SPSS Statistics version 24.0 (IBM Corp., Armonk, NY, USA) was used to perform all calculations, with two-sided *p* < 0.05 set as the statistically significant level.

## 5. Conclusions

In the GWAS of the TWB cohort, we found that three *RETN* lead SNPs (rs3219175, rs370006313, and rs3745368) were strongly associated with resistin levels and that two *IL1RL1* lead SNPs (rs10183388 and rs4142132) were significantly associated with sST2 levels in the Chinese population. These results were validated in the CH cohort and CAD cohort. Serum resistin and sST2 levels were significantly associated with cardiometabolic risk factors in the TWB and CAD cohorts and were strong predictors of poor clinical outcomes in patients with CAD. Furthermore, a synergistic effect was noted when combining resistin and sST2 levels with the WGRS of *RETN* and *IL1RL1* in the prognostication of CAD outcomes. Patients with high resistin levels/low *RETN* WGRS and high sST2 levels/low *IL1RL1* WGRS had the poorest outcome during long-term follow-up. This study provides insights into the effects of biomarkers and corresponding genetic variants on the prognosis of long-term clinical outcomes in patients with CAD.

## Figures and Tables

**Table 1:** Associations of *RETN* SNPs with resistin levels and *IL1RL1* SNPs with sST2 levels in the study populations.

Population	Genotypes	MAF	MM	Mm	mm	*β*	*p*1 Value	*p*2 Value
*RETN* SNPs and resistin levels								
TWB cohort (N = 4652)	rs3219175	0.188	10.1 (7.7–13.0) (3058)	19.6 (15.8–24.6) (1442)	32.4 (27.2–41.1) (152)	0.274	<1.00 × 10^−307^	<1.00 × 10^−307^
	rs370006313	0.009	12.3 (8.8 –18.5) (4570)	65.3 (49.5–89.0) (81)	43.1 (1)	0.676	2.70 × 10^−135^	8.04 × 10^−136^
	rs3745368	0.150	13.4 (9.7–19.8) (3377)	10.3 (7.3–16.3) (1151)	6.4 (4.5–10.1) (124)	−0.099	5.90 × 10^−42^	8.69 × 10^−42^
	*RETN* WGRS					1.062	<1.00 × 10^−307^	<1.00 × 10^−307^
CH cohort (N = 557)	rs3219175	0.203	12.40 (9.15–15.95) (361)	22.50 (17.48–29.75) (166)	39.30 (29.10–49.08) (30)	0.234	3.36 × 10^−41^	6.77 × 10^−41^
	rs370006313	0.006	14.8 (10.3–22.6) (550)	58.3 (54.7–86.5) (7)	--	0.685	2.42 × 10^−12^	1.36 × 10^−12^
	rs3745368	0.129	16.0 (11.20 –24.0) (413)	12.10 (8.2–18.8) (134)	7.90 (7.1–36.6) (5)	−0.100	2.80 × 10^−5^	2.70 × 10^−5^
	*RETN* WGRS					1.034	2.61 × 10^−65^	1.98 × 10^−65^
CAD cohort (N = 512)	rs3219175	0.194	11.1 (7.5–21.7) (333)	21.5 (13.1–39.4) (159)	27.0 (18.4–42.3) (20)	0.211	4.44 × 10^−15^	4.89 × 10^−15^
	rs370006313	0.008	15.2 (8.7–26.7) (504)	40.9 (16.7–52.9) (8)	--	0.263	0.035	0.055
	rs3745368	0.159	16.3 (9.3–30.3) (365)	12.4 (7.2–22.5) (131)	13.4 (6.4–24.3) (16)	−0.093	0.001	0.001
	*RETN* WGRS					1.041	1.69 × 10^−19^	1.84 × 10^−19^
*IL1RL1* SNPs and sST2 level								
TWB cohort (N = 4652)	rs10183388	0.456	4.9 (3.6–6.5) (1370)	7.6 (5.9–10.0) (2352)	9.9 (7.5–12.9) (957)	0.157	6.16 × 10^−294^	<1.00 × 10^−307^
	rs4142132	0.491	5.2 (3.9–6.8) (1180)	7.4 (5.6–9.8) (2375)	9.3 (7.1–12.5) (1097)	0.125	1.23 × 10^−176^	1.92 × 10^−212^
	*IL1RL1*-WGRS					0.520	1.15 × 10^−246^	3.71 × 10^−296^
CH cohort (N = 557)	rs10183388	0.435	7.4 (5.3–9.5) (179)	10.2 (8.0–13.2) (271)	13.2 (8.8–18.3) (107)	0.130	2.93 × 10^−14^	9.11 × 10^−16^
	rs4142132	0.480	7.6 (5.8–9.5) (154)	9.9 (7.5–13.2) (271)	11.7 (8.2–16.6) (132)	0.090	1.48 × 10^−7^	2.73 × 10^−8^
	*IL1RL1*-WGRS					0.547	7.67 × 10^−12^	4.56 × 10^−13^
CAD cohort (N = 512)	rs10183388	0.457	6.1 (3.4–8.7) (162)	6.4 (4.6–9.3) (234)	8.6 (6.0–12.4) (116)	0.092	2.64 × 10^−8^	4.49 × 10^−8^
	rs4142132	0.496	6.0 (3.7–8.8) (133)	6.4 (4.4–9.3) (250)	8.3 (5.9–12.2) (129)	0.077	5.00 × 10^−6^	8.00 × 10^−6^
	*IL1RL1*-WGRS					0.533	1.83 × 10^−7^	3.01 × 10^−7^

TWB, Taiwan Biobank; CH, cardiovascular healthy examination; CAD, cardiovascular disease; MAF, minor allele frequency; M, major allele; m, minor allele; sST2, soluble suppression of tumorigenicity 2; WGRS, weighted genetic risk score. *p*1: unadjusted, *p*2: adjusted for age, sex, body mass index, and current smoking status.

**Table 2:** Baseline characteristics between TWB, CH, and CAD groups.

	TWB (N = 4652)	CH (N = 557)	CAD (N = 512)
Age	48.6 ± 11.0	46.0 ± 9.9 ^b^	65.5 ± 11.1 ^b,c^
Male sex ^§^	2067 (44.4%)	290 (52.1%)	413 (80.7%)
Smoking ^§^	861 (18.5%)	107 (9.2%)	126 (24.6%)
Obesity ^§^	1692 (36.4%)	225 (40.4%)	301 (58.8%)
Hypertension ^§^	826 (17.8%)	110 (19.7%)	400 (78.1%)
Diabetes mellitus ^§^	264 (5.7%)	28 (5.0%)	228 (44.5%)
Dyslipidemia ^§^	2197 (47.2%)	297 (53.3%)	329 (64.3%)
BMI (kgw/m^2^) ^§^	24.2 ± 3.6	24.4 ± 3.4	26.1 ± 4.0 ^b,c^
Fasting glucose (mmol/L)	5.29 ± 1.04	5.41 ± 1.35	6.30 ± 2.04 ^b,c^
Cholesterol (mmol/L)	5.03 ± 0.93	5.14 ± 0.94 ^a^	4.63 ± 1.01 ^b,c^
Triglyceride (mmol/L) *	1.05 (0.73–1.54)	1.32 (0.89–1.88) ^b^	1.40 (1.01–2.04) ^b^
HDL-C (mmol/L)	1.41 ± 0.34	1.42 ± 0.36	1.02 ± 0.40 ^b,c^
LDL-C (mmol/L)	3.14 ± 0.82	3.00 ± 0.84 ^b^	2.61 ± 0.81 ^b,c^
AST (μkat/L)	0.41 ± 0.25	-	0.43 ± 0.27 ^a^
Uric acid (μmol/L)	331.9 ± 87.4	379.0 ± 95.8 ^b^	387.2 ± 105.7 ^b^
Creatinin (μmol/L)	65.4 ± 17.7	87.5 ± 42.4 ^b^	122.9 ± 125.5 ^b,c^
eGFR (mL/min/1.73 m^2^)	95.0 ± 20.3	84.2 ± 20.6 ^b^	65.7 ± 22.9 ^b,c^
Leukocyte count (10^3^/uL)	6.02 ± 1.57	-	6.67 ± 2.12 ^b^
Hematocrit (%)	43.3 ± 4.58	-	40.8 ± 5.38 ^b^
Platelet counts (10^3^/uL)	239.8 ± 56.6	-	214.5 ± 62.9 ^b^
Resistin (ng/mL) *	12.44 (8.86–18.95)	14.90 (10.35–22.9) ^b^	15.39 (8.68–26.98) ^b^
sST2 (ng/mL) *	7.12 (5.14–9.84)	9.33 (7.09–12.94) ^b^	6.76 (4.49–9.63) ^c^

Continuous data are presented as the mean ± SD or median (interquartile range), and categorical data are presented as numbers (%). Abbreviations: AST, aspartate aminotransferase; BMI, body mass index; CAD, cardiovascular disease; CH, cardiovascular healthy examination; eGFR, estimated glomerular filtration rate; HDL, high-density lipoprotein cholesterol; LDL-C, low-density lipoprotein cholesterol; TWB, Taiwan Biobank. AST, leukocyte count, hematocrit, and platelet counts were not available in the CH cohort. * Data with skew distribution are logarithmically transformed before statistical testing to meet the assumption of normal distribution. ^§^ *p* < 0.01 for χ2 test. ^a^ *p* < 0.05 vs. Q1 by the Bonferroni method. ^b^ *p* < 0.001 vs. Q1 by Bonferroni method. ^c^ *p* < 0.001 vs. Q2 by the Bonferroni method.

**Table 3:** Cox regression analysis of all-cause mortality and MACEs rate between the groups stratified by the presence of high resistin level/low *RETN* WGRS and high sST2 levels/low *IL1RL1* WGRS.

	Group 1 *	Group 2 *		Group 3 *	
All-cause mortality					
Patient numbers	334	158		20	
Number of events	7	17		7	
		HR (95% CI)	*p*-value	HR (95% CI)	*p*-value
Model 1	Reference	5.04 (2.09–12.15)	3.22 × 10^−4^	19.59 (6.86–55.91)	2.71 × 10^−8^
Model 2	Reference	5.04 (2.08–12.22)	3.48 × 10^−4^	15.21 (5.24–44.13)	5.48 × 10^−7^
Model 3	Reference	5.14 (2.10–12.55)	3.29 × 10^−4^	11.59 (3.82–35.17)	1.50 × 10^−5^
Model 4	Reference	3.78 (1.08–13.23)	0.037	21.76 (4.50–105.11)	1.27 × 10^−4^
MACEs					
Patient numbers	334	158		20	
Number of events	23	28		7	
		HR (95% CI)	*p*-value	HR (95% CI)	*p*-value
Model 1	Reference	2.62 (1.51–4.55)	0.001	5.88 (2.52–13.71)	4.1 × 10^−5^
Model 2	Reference	2.70 (1.55–4.69)	4.50 × 10^−4^	5.42 (2.30–12.78)	1.11 × 10^−4^
Model 3	Reference	2.74 (1.57–4.78)	4.10 × 10^−4^	4.87 (2.06–11.56)	3.24 × 10^−4^
Model 4	Reference	1.72 (0.82–3.64)	0.153	4.03 (1.29–12.59)	0.017

* Group 1, patients without high resistin levels/low *RETN* WGRS and high sST2 levels/low *IL1RL1* WGRS; Group 2, patients with either high resistin levels/low *RETN* WGRS or high sST2 levels/low *IL1RL1* WGRS; Group 3, patients with both high resistin levels/low *RETN* WGRS and high sST2 levels/low *IL1RL1* WGRS. Abbreviations: HR, hazard ratio; CI, confidence interval. Model 1, Unadjusted. Model 2, Adjusted for age, sex, BMI, and smoking status. Model 3: Adjusted for age, sex, BMI, smoking status, diabetes mellitus, hypertension, and dyslipidemia. Model 4: Adjusted for age, sex, BMI, smoking status, diabetes mellitus, hypertension, dyslipidemia, uric acid level, estimated glomerular filtration rate, CRP levels, chemerin levels, and GDF-15 levels.

## Data Availability

The data presented in this study are available on request from the corresponding author.

## Supplementary Materials

The following supporting information can be downloaded at: https://www.mdpi.com/article/10.3390/ijms23084292/s1.
