# Supplementary material for: Synergistic Effects of Weighted Genetic Risk Scores and Resistin and sST2 Levels on the Prognostication of Long-Term Outcomes in Patients with Coronary Artery Disease
Source: Int J Mol Sci. 2022 Apr 13;23(8):4292. doi: 10.3390/ijms23084292 (PMC9025936; doi:10.3390/ijms23084292)
Supplement: Supplementary file 1 [file ijms-23-04292-s001.zip › ijms-1667024-supplementary.pdf]

Supplementary Materials

Synergistic effects of weighted genetic risk scores and resistin and sST2 levels on the prognostication of long-term outcomes in patients with coronary artery disease

Supplementary Figure S1. Regional association plots for resistin levels from the whole genome sequence study of 880 Taiwan Biobank participants with 509 SNPs at positions between 7.715 and 7.755 Mb on chromosome 19p13.2 around the *RETN* gene region.

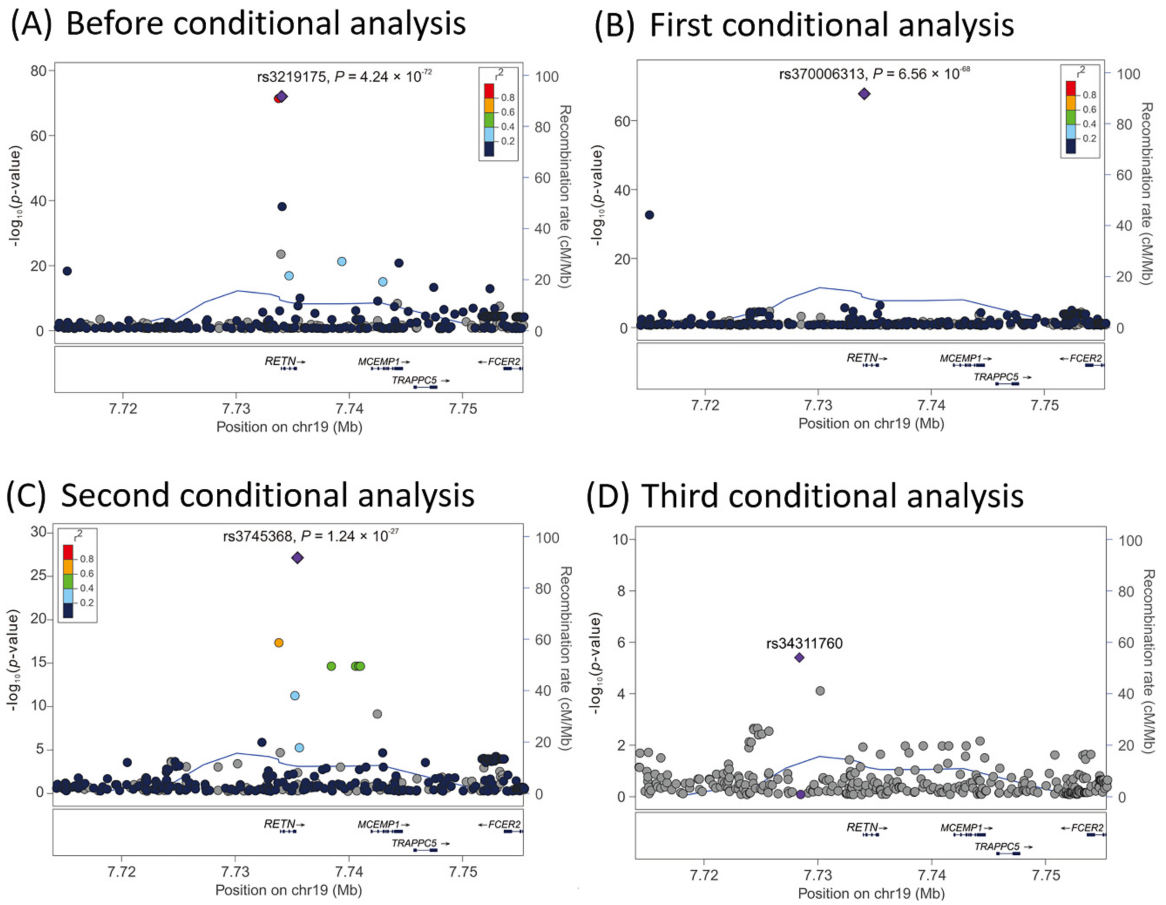

(A) Before conditional analysis, regional association plots show rs3219175 as the lead SNP that is strongly associated with resistin levels. (B) After first conditional analysis adjusting for rs3219175 genotype, rs370006313 in the regional plot of *RETN* locus becomes significant association with resistin levels (C) After second conditional analysis adjusting for both rs3219175 and rs370006313 genotypes, rs3745368 is significantly associated with resistin levels. (D) After third conditional analysis adjusting for the aforementioned SNPs, no more single SNP is found to be genome-wide significantly associated with resistin levels.

Supplementary Figure S2. Regional association plots for sST2 levels from the whole genome sequence study of 859 Taiwan Biobank participants with 307 SNPs at positions between 102.915 and 102.985 Mb on chromosome 2q12.1 around the *IL1RL1* gene region.

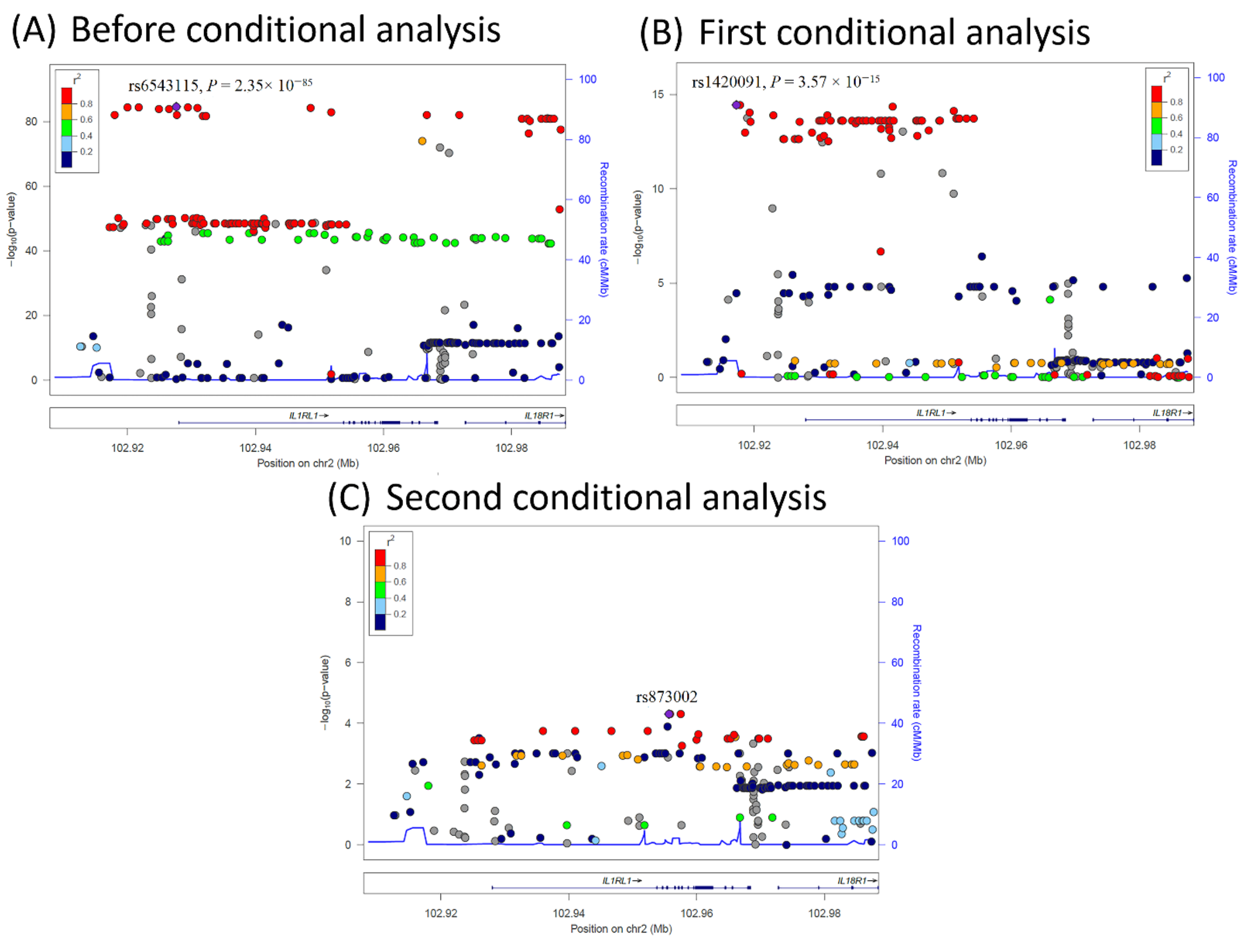

(A) Before conditional analysis, regional association plots show rs6543115 as the lead SNP that is strongly associated with sST2 levels. (B) After first conditional analysis adjusting for rs6543115 genotype, rs1420091 in the regional plot at the *IL1RL1* locus became more significant association with sST2 levels. (C) After second conditional analysis adjusting for the two aforementioned SNPs, none of the SNP is found to be genome-wide significantly associated with sST2 levels.

Supplementary Figure S3. Linkage dysequilibrium of *IL1RL1* lead SNPs.

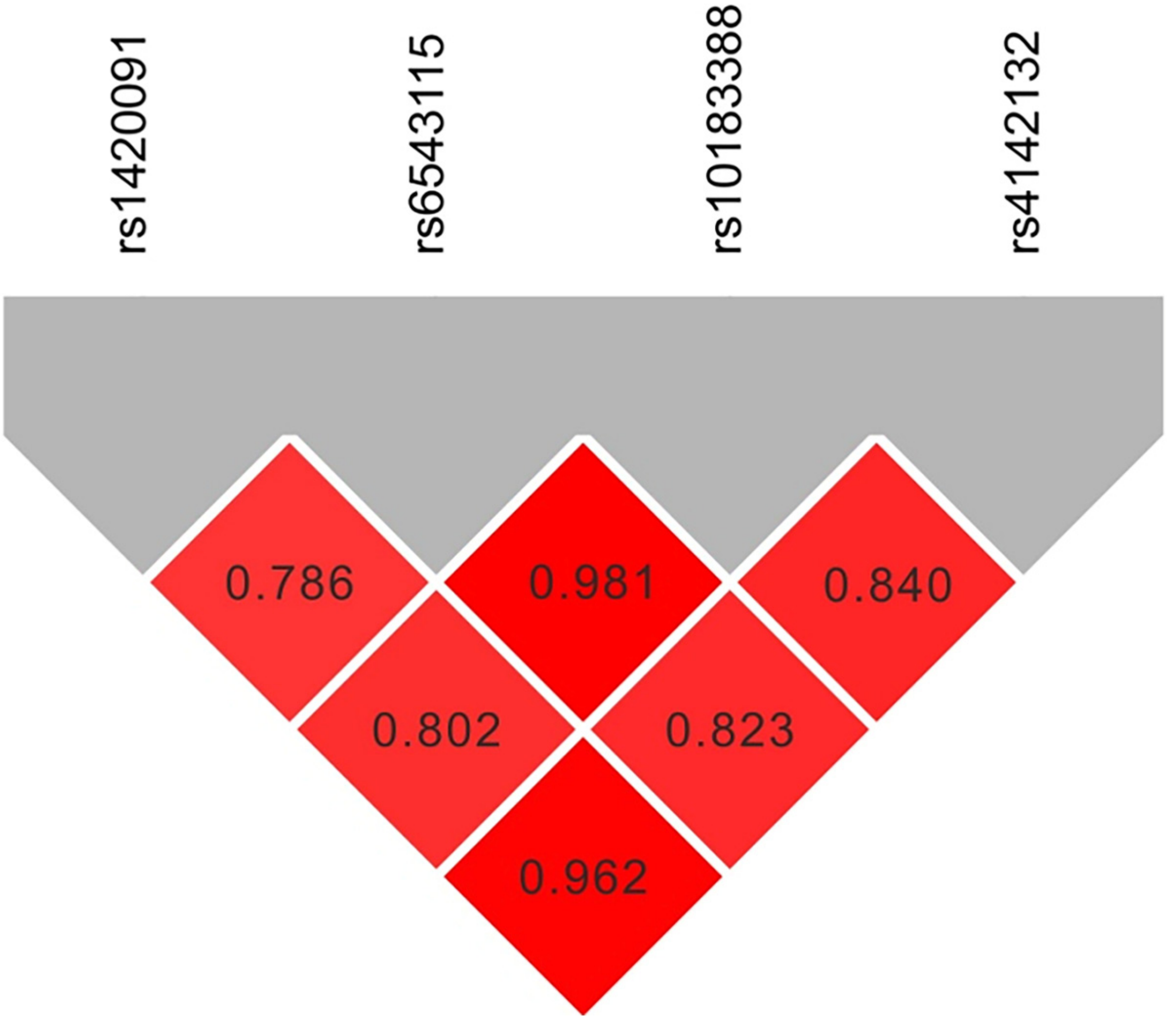

The *IL1RL1* lead SNPs rs10183388 and rs4142132 from the GWAS study of Taiwan Biobank participants are in nearly complete linkage disequilibrium with the *IL1RL1* lead SNPs rs6543115 and rs1420091 respectively in the WGS study (r^2 = 0.98 and 0.96, respectively).

Supplementary Figure S4. The flowchart of participants enrolled in the (A) Taiwan Biobank cohort (B) Cardiovascular healthy examination cohort and (C) coronary artery disease cohort.

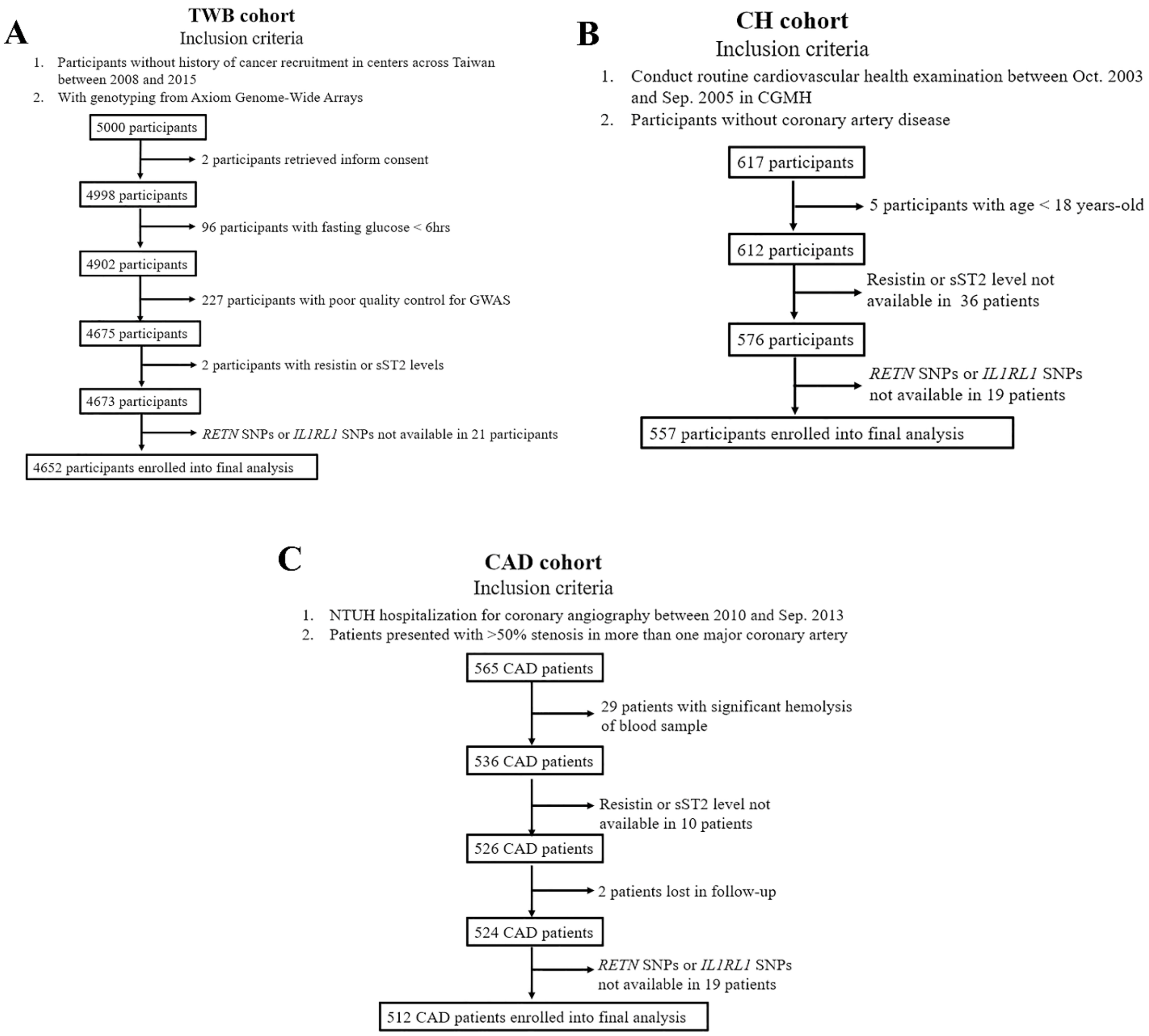

Supplementary Table S1. Stepwise linear regression for the resistin and sST2 levels in Taiwan Biobank cohort

	Resistin level				sST2 levels			
	Beta	se	r^2	*P*	Beta	se	r^2	*P*
Sex	-0.034	0.005	0.006	2.21×10^{-10}	-0.1626	0.005	0.136	3.90×10^{-212}
Age	--	--	--	--	0.001	<0.001	0.001	0.002
Body mass index	0.003	0.001	0.001	4.83×10^{-4}	--	--	--	--
Smoking	--	--	--	--	--	--	--	--
*RETN*rs3219175	0.272	0.005	0.339	$<1.00\times10^{-307}$				
*RETN*rs370006313	0.828	0.020	0.138	$<1.00\times10^{-307}$				
*RETN*rs3745368	-0.126	0.005	0.060	6.01×10^{-126}				
*IL1RL1*rs10183388					0.290	0.009	0.251	4.36×10^{-191}
*IL1RL1*rs4142132					-0.143	0.009	0.029	7.93×10^{-51}

sST2, soluble suppression of tumorigenicity 2.

Supplementary Table S2. SNPs table of *RETN* and *IL1RL1*

Chr	rs number	Position	Gene locus	Location related to TIS	Function	Genotype	MAF
19	rs3219175	19:7733855	*RETN*	-358	TFBS	G/A	0.188
19	rs370006313	19:7733894	*RETN*	-319	--	G/A	0.009
19	rs3745368	19:7735297	*RETN*	1084	Splicing(ESE or ESS)、miRNA	G/A	0.150
2	rs10183388	102932247	*IL1RL1*	4285	Intron Variant	T/C	0.456
2	rs4142132	102937482	*IL1RL1*	9520	Intron Variant	G/A	0.4891

SNP, single nucleotide polymorphism; MAF, minor allele frequency; TFBS, transcription factor binding site; TIS, transcription initiation site.

Supplementary Table S3. Correlation of resistin and sST2 levels with clinical, metabolic, biochemical and hematological parameters in Taiwan Biobank participants (N=4652)

	Resistin levels*				sST2 levels*			
	Model 1		Model 2		Model 1		Model 2	
	Standard β	P value	Standard β	P value	Standard β	P value	P value	P value
Age	-0.031	0.032	-	-	0.024	0.108	-	-
Female	-0.078	1.09×10^{-7}	-	-	-0.366	1.31×10^{-147}	-	-
BMI	0.053	3.03×10^{-4}	-	-	0.084	9.33×10^{-9}	-	-
Smoking	0.056	1.19×10^{-4}	-	-	0.106	3.49×10^{-13}	-	-
Hypertension	0.029	0.048	0.025	0.107	0.062	2.50×10^{-5}	0.013	0.376
Diabetes	0.035	0.017	0.033	0.027	0.060	7.00×10^{-6}	0.048	0.001
Cholesterol	-0.063	1.8×10^{-5}	-0.061	3.9×10^{-5}	0.027	0.069	0.038	0.006
Triglyceride*	0.037	0.011	0.012	0.462	0.100	7.14×10^{-12}	0.016	0.283
HDL-C	-0.089	1.46×10^{-9}	-0.063	1.24×10^{-4}	-0.096	5.66×10^{-11}	0.044	0.005
LDL-C	-0.039	0.007	-0.047	0.002	0.039	0.007	0.019	0.168
AST	0.025	0.085	0.033	0.332	0.156	1.67×10^{-26}	0.116	7.12×10^{-17}
Uric acid	0.055	1.78×10^{-4}	0.006	0.761	0.218	3.21×10^{-51}	0.038	0.028
eGFR	-0.087	2.35×10^{-9}	-0.090	1.50×10^{-8}	-0.133	6.49×10^{-20}	-0.037	0.014
Leukocyte counts	0.133	9.00×10^{-20}	0.121	1.81×10^{-15}	0.107	2.19×10^{-13}	0.083	5.46×10^{-9}
Hematocrit	0.057	1.10×10^{-4}	0.009	0.608	0.223	1.30×10^{-53}	0.005	0.780
Platelet counts	0.023	0.111	0.033	0.034	-0.079	8.15×10^{-8}	0.011	0.451
RETN WGRS	0.729	0.00E0	0.782	0.00E0				
IL1RL1 WGRS					0.464	1.15×10^{-246}	0.524	3.71×10^{-296}

sST2, soluble suppression of tumorigenicity 2; BMI, body mass index; HDL-C, high-density lipoprotein cholesterol; LDL-C, low-density lipoprotein cholesterol; AST, aspartate aminotransferase; eGFR, estimated glomerular filtration rate; WGRS, weighted genetic risk score

*Data with skew distribution are logarithmically transformed before statistical testing to meet the assumption of normal distribution.

Model 1: Unadjusted correlations between resistin and sST2 levels with variables of interest.

Model 2: Adjusted for age, sex, BMI and smoking.

Supplementary Table S4. Correlation of resistin and sST2 levels with clinical, metabolic, biochemical and hematological parameters in CH participants (N=557)

	Resistin levels*				sST2 levels*			
	Model 1		Model 2		Model 1		Model 2	
	Standard β	P value	Standard β	P value	Standard β	P value	Standard β	P value
Age	0.023	0.581	-	-	-0.028	0.530	-	-
Female	0.054	0.207	-	-	-0.258	6.59×10^{-10}	-	-
-0.003	0.003	0.946	-	-	0.006	0.889	-	-
Smoking	0.018	0.680	-	-	0.082	0.052	-	-
Hypertension	-0.004	0.927	-0.013	0.783	0.005	0.914	0.009	0.840
Diabetes	0.036	0.402	0.035	0.409	0.014	0.733	0.012	0.765
Fasting glucose	0.013	0.767	0.016	0.378	0.033	0.434	0.012	0.768
Cholesterol	-0.103	0.015	-0.110	0.011	0.041	0.336	0.036	0.394
Triglyceride*	0.003	0.936	0.007	0.877	0.046	0.276	-0.022	0.637
HDL-C	-0.023	0.073	-0.053	0.275	-0.096	0.023	-0.005	0.919
LDL-C	-0.095	0.025	-0.096	0.025	0.071	0.095	0.060	0.149
Uric acid	-0.056	0.230	-0.031	0.580	0.093	0.044	-0.023	0.668
eGFR	-0.066	0.157	-0.015	0.149	0.030	0.516	0.017	0.808
RETN WGRS	0.639	2.61×10^{-65}	0.641	1.98×10^{-65}				
IL1RL1 WGRS					0.261	3.72×10^{-10}	0.266	5.25×10^{-11}

CH, Cardiovascular Health examination; BMI, body mass index; HDL-C, high-density lipoprotein cholesterol; LDL-C, low-density lipoprotein cholesterol; eGFR, estimated glomerular filtration rate; WGRS, weighted genetic risk score

*Data with skew distribution are logarithmically transformed before statistical testing to meet the assumption of normal distribution.

Model 1: Unadjusted correlations between resistin and sST2 levels with variables of interest.

Model 2: Adjusted for age, sex, BMI and smoking.

Supplementary Table S5. Correlation of resistin and sST2 levels with clinical, metabolic, biochemical and hematological parameters in CAD participants (N=512)

	Resistin levels*				sST2 levels*			
	Model 1		Model 2		Model 1		Model 2	
	Standard β	P value	Standard β	P value	Standard β	P value	Standard β	P value
Age	0.004	0.932	-	-	0.053	0.229	-	-
Female	0.096	0.030			-0.001	0.978	-	-
BMI	0.013	0.775			-0.035	0.428	-	-
Smoking	0.011	0.801			0.050	0.259	-	-
Hypertension	0.004	0.926			-0.003	0.946	0.004	0.936
Diabetes	0.095	0.031	0.080	0.076	0.082	0.065	0.085	0.061
Fasting glucose	-0.045	0.358	-0.058	0.245	0.020	0.684	0.027	0.583
Cholesterol	0.071	0.110	0.060	0.184	0.025	0.573	0.021	0.645
Triglyceride*	0.058	0.191	0.044	0.350	-0.026	0.564	-0.014	0.773
HDL-C	0.024	0.780	0.002	0.980	0.009	0.919	-0.039	0.675
LDL-C	0.084	0.337	0.118	0.185	0.123	0.155	0.105	0.239
AST	0.007	0.874	0.003	0.955	0.092	0.047	0.091	0.050
Uric acid	-0.040	0.391	-0.039	0.405	-0.007	0.887	-0.014	0.774
eGFR	-0.170	1.35×10^{-4}	-0.174	1.76×10^{-4}	-0.194	1.30×10^{-5}	-0.194	2.80×10^{-5}
Leukocyte counts	0.183	3.40×10^{-5}	0.182	7.20×10^{-5}	0.135	0.002	0.144	0.002
Hematocrit	-0.132	0.003	-0.137	0.006	-0.166	1.71×10^{-4}	-0.193	1.19×10^{-4}
Platelet counts	0.072	0.104	0.062	0.189	-0.045	0.312	-0.036	0.447
RETN WGRS	0.385	1.09×10^{-19}	0.385	1.84×10^{-19}				
IL1RL1 WGRS					0.228	1.83×10^{-7}	0.226	3.01×10^{-7}

CAD, coronary artery disease; BMI, body mass index; HDL-C, high-density lipoprotein cholesterol; LDL-C, low-density lipoprotein cholesterol; AST, aspartate aminotransferase; eGFR, estimated glomerular filtration rate; WGRS, weighted genetic risk score

*Data with skew distribution are logarithmically transformed before statistical testing to meet the assumption of normal distribution.

Model 1: Unadjusted correlations between resistin and sST2 levels with variables of interest.

Model 2: Adjusted for age, sex, BMI and smoking.

Supplementary Table S6. Cox regression analysis of all-cause mortality and MACEs rate between the groups stratified by the resistin levels and *RETN* WGRS

	Group 1*	Group 2*		Group 3*		Group 4*	
All-cause mortality							
Patient numbers	201	55		132		124	
Number of events	5	0		6		20	
		HR (95% CI)	P value	HR (95% CI)	P value	HR (95% CI)	P value
Model 1	Reference	0 (0)	0.980	1.72 (0.53-5.64)	0.371	6.24 (2.34-16.65)	2.55×10^{-4}
Model 2	Reference	0 (0)	0.973	1.41 (0.43-4.66)	0.573	5.83 (2.19-15.62)	4.34×10^{-4}
Model 3	Reference	0 (0)	0.980	1.42 (0.43-4.70)	0.565	5.53 (2.07-14.78)	6.52×10^{-4}
Model 4	Reference	0 (0)	0.979	1.65 (0.29-9.58)	0.574	7.44 (1.52-36.44)	0.013
MACEs							
Patient numbers	201	55		132		124	
Number of events	14	4		6		20	
		HR (95% CI)	P value	HR (95% CI)	P value	HR (95% CI)	P value
Model 1	Reference	1.11 (0.37-3.38)	0.835	1.34 (0.63-2.85)	0.453	3.08 (1.61-5.88)	6.47×10^{-4}
Model 2	Reference	1.14 (0.37-3.46)	0.823	1.19 (0.56-2.54)	0.656	3.05 (1.60-5.84)	7.35×10^{-4}
Model 3	Reference	0.99 (0.32-3.04)	0.984	1.17 (0.42-1.99)	0.686	2.97 (1.55-5.68)	9.87×10^{-4}
Model 4	Reference	1.07 (0.35-3.28)	0.912	0.91 (0.42-1.99)	0.820	1.62 (0.78-3.35)	0.197

*Group 1, low resistin levels/low *RETN* WGRS; Group 2, low resistin levels/high *RETN* WGRS; Group 3, high resistin levels/high *RETN* WGRS; Group 4, high resistin levels/low *RETN* WGRS.

Abbreviations: HR, hazard ration; CI, confidence interval

Model 1, Unadjusted

Model 2, Adjusted for age, sex, BMI and smoking status

Model 3: Adjusted for age, sex, BMI, smoking status, diabetes mellitus, hypertension, and dyslipidemia.

Model 4: Adjusted for age, sex, BMI, smoking status, diabetes mellitus, hypertension, dyslipidemia, uric acid level, estimated glomerular filtration rate, CRP levels, chemerin levels and GDF-15 levels

Supplementary Table S7. Cox regression analysis of all-cause mortality and MACEs rate between the groups stratified by the sST2 levels and *IL1RL1* WGRS

	Group 1*	Group 2*		Group 3*		Group 4*	
All-cause mortality							
Patient numbers	86	170		182		74	
Number of events	1	7		12		11	
		HR (95% CI)	P value	HR (95% CI)	P value	HR (95% CI)	P value
Model 1	Reference	3.75 (0.46-30.45)	0.217	5.60 (0.73-43.10)	0.098	14.13 (1.82-109.55)	0.011
Model 2	Reference	3.80 (0.47-30.95)	0.213	5.76 (0.74-44.72)	0.094	12.44 (1.59-97.08)	0.016
Model 3	Reference	3.60 (0.44-29.44)	0.233	5.49 (0.71-42.74)	0.104	10.55 (1.32-84.02)	0.026
Model 4	Reference	2.61 (0.29-23.24)	0.389	1.46 (0.16-13.49)	0.739	4.99 (0.57-43.61)	0.147
MACEs							
Patient numbers	86	170		182		74	
Number of events	3	16		24		15	
		HR (95% CI)	P value	HR (95% CI)	P value	HR (95% CI)	P value
Model 1	Reference	2.93 (0.85-10.04)	0.088	3.04 (0.91-10.16)	0.071	6.04 (1.75-20.93)	0.005
Model 2	Reference	2.91 (0.85-10.00)	0.091	3.08 (0.92-10.34)	0.069	6.08 (1.75-21.12)	0.005
Model 3	Reference	3.00 (0.87-10.38)	0.082	3.04 (0.90-10.24)	0.073	5.90 (1.68-20.72)	0.006
Model 4	Reference	2.70 (0.73-9.97)	0.136	1.73 (0.48-6.31)	0.406	3.24 (0.81-12.88)	0.095

*Group 1, low sST2 levels/low *IL1RL1* WGRS; Group 2, low sST2 levels/high *IL1RL1* WGRS; Group 3, high sST2 levels/high *IL1RL1* WGRS; Group 4, high sST2 levels/low *IL1RL1* WGRS.

Abbreviations: HR, hazard ration; CI, confidence interval

Model 1, Unadjusted

Model 2, Adjusted for age, sex, BMI and smoking status

Model 3: Adjusted for age, sex, BMI, smoking status, diabetes mellitus, hypertension, and dyslipidemia.

Model 4: Adjusted for age, sex, BMI, smoking status, diabetes mellitus, hypertension, dyslipidemia, uric acid level, estimated glomerular filtration rate, CRP levels, chemerin levels and GDF-15 levels

Supplementary Table S8. The inflammatory biomarker levels according to the resistin level and *RETN* WGRS and sST2 levels and *IL1RL1* WGRS

Resistin level and *RETN* WGRS						
	Low resistin levels Low *RETN* WGRS	Low resistin levels High *RETN* WGRS	High resistin levels High *RETN* WGRS	High resistin levels Low *RETN* WGRS	P	P1
Uric acid	396.8±106.5	371.2±97.0	373.6±104.1	395.0±109.5	0.153	0.213
eGFR	68.3±19.7	75.8±20.1	66.3±22.2	55.5±26.3	2.37×10^{-8}	0.018
CRP	2.20 (1.10-3.90)	2.35 (1.48-3.33)	2.35 (1.13-4.28)	3.10 (1.70-5.90)	0.059	0.053
Chemerin	116.1 (91.9-150.1)	99.9 (80.6-131.1)	134.8 (102.7-166.5)	145.5 (117.3-201.7)	3.00×10^{-6}	5.00×10^{-6}
GDF-15	0.85 (0.61-1.20)	0.74 (0.56-1.10)	1.10 (0.79-1.76)	1.26 (0.81-2.47)	1.56×10^{-8}	1.35×10^{-8}
sST2 level and *IL1RL1* WGRS						
	Low sST2 levels Low *IL1RL1* WGRS	Low sST2 levels High *IL1RL1* WGRS	High sST2 levels High *IL1RL1* WGRS	High sST2 levels Low *IL1RL1* WGRS	P	P1
Uric acid	388.9±104.8	388.2±105.0	390.8±110.6	375.9±97.6	0.828	0.999
eGFR	67.6±23.1	69.9±19.8	62.3±23.5	62.0±23.5	0.008	0.004
CRP	2.00 (1.28-3.20)	2.60 (1.43-4.58)	2.60 (1.15-5.10)	2.80 (1.48-5.10)	0.084	0.126
Chemerin	121.3 (91.6-147.4)	120.6 (93.0-151.5)	135.6 (98.9-175.2)	133.2 (102.0-171.8)	0.001	2.60×10^{-4}
GDF-15	0.88 (0.63-1.35)	0.87 (0.68-1.16)	1.06 (0.73-2.08)	1.30 (0.70-2.22)	8.9×10^{-5}	6.60×10^{-8}
Combined High resistin levels/low *RETN* WGRS group and high sST2 levels/low *IL1RL1* WGRS group						
	None of these two groups	Either one of these two groups	Both of these two groups		P	P1
Uric acid	386.7±105.3	391.4±107.7	370.0±94.0		0.729	0.639
eGFR	69.2±20.4	60.4±25.0	47.2±30.0		9.07×10^{-7}	2.00×10^{-6}
CRP	2.30 (1.20-3.87)	2.90 (1.45-5.80)	3.50 (2.10-5.48)		0.007	0.006
Chemerin	121.1 (91.3-153.3)	137.3 (102.9-179.2)	171.7 (118.5-1256.1)		6.72×10^{-8}	4.12×10^{-7}
GDF-15	0.88 (0.65-1.33)	1.14 (0.77-2.02)	1.94 (0.99-4.30)		5.40×10^{-10}	4.21×10^{-10}

sST2, sST2, soluble suppression of tumorigenicity 2; WGRS, weighted genetic risk scores; eGFR, estimated glomerular filtration rate; CRP, C reactive protein; GDF-15, growth differentiation factor 15.

P1; adjusted for age, sex, body mass index and smoking

Supplementary Table S9. Definition of disease and smoking status

Hypertension	Patients were considered to have hypertension if they had systolic blood pressure (BP) ≥ 140 mm Hg, diastolic BP ≥ 90 mm Hg or medical records documenting hypertension and the prescription of antihypertensive drugs.
Diabetes mellitus	DM was defined according to the guidelines of the American Diabetes Association as a fasting plasma glucose level ≥ 126 mg/dL, a glycohemoglobin value ≥ 6.5%, or having medical records documenting diabetes mellitus and receiving hypoglycemic agents.
Dyslipidemia	Dyslipidemia was defined as serum cholesterol level ≥200 mg/dL, triglyceride level ≥200 mg/dL or the use of lipid-lowering drugs.
Smoking	Smoking was determined based on history, previous medical records, current medication, and by examination during hospitalization. Current smokers were defined as those who regularly smoked cigarettes at the time of the survey.

Supplementary Table S10. Biomarkers inter- and intra-assay variability measures

Biomarkers	Source	Intra-assay	Inter-assay
Resistin	Plasma	CV=3.8%	CV=5.8%
sST2	Plasma	CV=4.9%	CV=5.0%
CRP	Serum	CV=7.1%	CV=9.5%
Chemerin	Plasma	CV=1.4%	CV=4.2%
GDF-15	PLasma	CV=1.2%	CV=5.5%

sST2, soluble suppression of tumorigenicity 2; CRP, C-reactive protein; GDF, growth differentiation factor;

CV: coefficient of variation.
